# Discovery and identification of potential biomarkers of pediatric Acute Lymphoblastic Leukemia

**DOI:** 10.1186/1477-5956-7-7

**Published:** 2009-03-16

**Authors:** Linan Shi, Jun Zhang, Peng Wu, Kai Feng, Jing Li, Zhensheng Xie, Peng Xue, Tanxi Cai, Ziyou Cui, Xiulan Chen, Junjie Hou, Jianzhong Zhang, Fuquan Yang

**Affiliations:** 1 Proteomic Platform, Institute of Biophysics, Chinese Academy of Sciences, Beijing 100101, PR China; 2 Graduate University of the Chinese Academy of Sciences, Beijing 100101, PR China; 3 Center for Experimental Medicine, 306 Hospital of PLA, Beijing 100101, PR China

## Abstract

**Background:**

Acute lymphoblastic leukemia (ALL) is a common form of cancer in children. Currently, bone marrow biopsy is used for diagnosis. Noninvasive biomarkers for the early diagnosis of pediatric ALL are urgently needed. The aim of this study was to discover potential protein biomarkers for pediatric ALL.

**Methods:**

Ninety-four pediatric ALL patients and 84 controls were randomly divided into a "training" set (45 ALL patients, 34 healthy controls) and a test set (49 ALL patients, 30 healthy controls and 30 pediatric acute myeloid leukemia (AML) patients). Serum proteomic profiles were measured using surface-enhanced laser desorption/ionization-time-of-flight mass spectroscopy (SELDI-TOF-MS). A classification model was established by Biomarker Pattern Software (BPS). Candidate protein biomarkers were purified by HPLC, identified by LC-MS/MS and validated using ProteinChip immunoassays.

**Results:**

A total of 7 protein peaks (9290 m/z, 7769 m/z, 15110 m/z, 7564 m/z, 4469 m/z, 8937 m/z, 8137 m/z) were found with differential expression levels in the sera of pediatric ALL patients and controls using SELDI-TOF-MS and then analyzed by BPS to construct a classification model in the "training" set. The sensitivity and specificity of the model were found to be 91.8%, and 90.0%, respectively, in the test set. Two candidate protein peaks (7769 and 9290 m/z) were found to be down-regulated in ALL patients, where these were identified as platelet factor 4 (PF4) and pro-platelet basic protein precursor (PBP). Two other candidate protein peaks (8137 and 8937 m/z) were found up-regulated in the sera of ALL patients, and these were identified as fragments of the complement component 3a (C3a).

**Conclusion:**

Platelet factor (PF4), connective tissue activating peptide III (CTAP-III) and two fragments of C3a may be potential protein biomarkers of pediatric ALL and used to distinguish pediatric ALL patients from healthy controls and pediatric AML patients. Further studies with additional populations or using pre-diagnostic sera are needed to confirm the importance of these findings as diagnostic markers of pediatric ALL.

## Introduction

Acute lymphoblastic leukemia (ALL) is the most common pediatric cancer, accounting for 30% of all pediatric malignancies [1]. ALL is diagnosed in 3000 to 4000 persons in the United States each year, where two-thirds of these are children [2,3]. ALL can develop from any lymphoid cell blocked at a particular stage of development, including primitive cells with multilineage potential [4,5]. The diagnosis of ALL presently depends on immunophenotyping. Although more than 339 different cluster-of-differentiation (CD) molecules expressed on human leukocytes are defined [6], only a few of these molecules are truly lineage-specific. Melhem reported that oncoprotein 18 (Op18) phosphorylation was significantly correlated with the white blood cell count and the percentage of cells in S phase [7]. However, Brattsand and Chen reported that over-phosphorylation of Op18 was also found in breast cancer and lung adenocarcinomas [8,9]. Noninvasive and specific biomarkers for early diagnoses of pediatric ALL remain an urgent need.

Proteome analysis provides valuable information about the total proteome's dynamic and rapid changes occurring during illness. Recent advances in proteomics have offered opportunities for finding biomarkers in biological fluids, especially in sera. Surface-assisted laser desorption/ionization time-of-flight mass spectrometry (SELDI-TOF-MS), which generates protein fingerprints, has been proven as a powerful tool for potential biomarker discovery [10,11]. Recently, SELDI-TOF-MS analysis has been successfully used to identify specific biomarkers for various cancers, such as prostate cancer, bladder cancer, ovarian cancer, lung cancer, colon cancer, breast cancer and pancreatic cancer [12-21]. Albitar explored the potential of proteomic analysis of peripheral blood plasma, using SELDI-TOF-MS, to predict the recurrence of ALL in adult patients and build a proteomic-based model predicting clinical behaviors in adult ALL [22]. Hegedus used SELDI-TOF-MS to analyze proteomes of pediatric leukemia cell lines, including ALL, mixed lineage leukemia (MLL) and acute myeloid leukemia (AML) cell lines, and pediatric leukemia bone marrow samples of different subtypes. Differences in protein expression were reported among ALL, MLL and AML cell lines and bone marrow. A protein of 8.3 kDa was found to be expressed at high levels in the ALL cell line as well as in bone marrow and was identified as a C-terminal truncated form of ubiquitin after purification and trypsin digestion. These results may guide further research toward understanding the development of leukemias [23].

In this study, we first used SELDI-TOF-MS technology to screen potential protein patterns specific to ALL, then purified the candidate protein biomarker peaks by HPLC and identified them by LC-MS/MS.

## Materials and methods

### Patients, controls and serum samples

The pediatric ALL patients were randomly selected from patients who had been confirmed by myelocytic cytological diagnosis in China-Japan Friendship Hospital during a given period of time (2004.7–2004.8). At the same time, the healthy controls were selected from children who took health examinations required for school or kindergarten entrance and had not been diagnosed with any other disease. Furthermore, the pediatric AML patients were also taken as controls from this hospital. Serum samples (n = 178) were collected from 94 ALL patients, ranging in ages from 2–14, 54 healthy children, and 30 pediatric acute myeloid leukemia (AML) patients. The three groups were similar in age and gender distribution (Table 1). All the serum samples were collected from the selected patients and controls in 2004. Informed consent was obtained from each participant. The collected sera were immediately centrifuged at 1500 × *g*, 4°C for 10 min after acquisition, then distributed into 20 μL/tube, and stored at -80°C for further analysis. All the SELDI-TOF-MS experiments were finished within three months.

**Table 1:** Age and sexual distribution of all study subjects in the SELDI-TOF-MS experiments

	Age (years)				Gender		total	Training set	Test set
	0–3	4–6	7–10	11–14	M (%)	F (%)			
ALL patients (M*/F**)	8/5	25/12	25/12	4/3	66.0%	34.0%	94	45	49
Healthy controls (M/F)	5/3	15/7	13/6	3/2	66.7%	33.3%	54	34	20
AML patients (M/F)	3/2	8/5	7/3	1/1	63.3%	36.7%	30		30
Total	16/10	48/24	45/21	8/6	65.7%	34.3%	178	79	99

M*: Male, F**: Female.

### SELDI-TOF-MS analysis of serum protein profiles

Protein profiling of serum samples was determined by SELDI-TOF-MS using the eight-spot format WCX2 (weak cation exchange) Proteinchip arrays (Ciphergen Biosystems, Fremont, CA, USA). Frozen serum samples were thawed on ice and spun at 10,000 rpm for 5 min at 4°C. Each serum sample (10 μL) was denatured by the addition of 20 μL of U9 buffer (9 M urea, 2% CHAPS, 50 mM Tris-HCl, 1% DTT, pH 9.0) and vortexed at 4°C for 30 min. Each sample was then diluted in 108 μL of low-stringency buffer (0.1 M sodium acetate, pH 4.0). At this point, 100 μL of each diluted serum sample were ready to hybridize with WCX2 Proteinchip arrays. The samples were held by a bioprocessor (Ciphergen Biosystems) and preactivated twice with 150 μL low stringency buffer at room temperature for 5 min. The diluted serum sample was allowed to react with the surface of the WCX2 chip for 60 min at room temperature. Each spot was then washed three times with appropriate buffers of various pHs and ionic strengths to eliminate non-adsorbed proteins. After drying the array surface in the air, 1 μL of saturated sinapinic acid (SA) matrix in 50% ACN and 0.5% TFA was applied and allowed to dry. MS analysis was performed on a PBS-II ProteinChip reader (Ciphergen Biosystems). Mass peak detection was analyzed using ProteinChip Biomarker Software version 3.1 (Ciphergen Biosystems). The mass spectra of the proteins were generated using an average of 90 laser shots at a laser intensity of 150–160 arbitrary units, and detector sensitivity was set at 8. For data acquisition of low-molecular weight proteins, the optimized detection mass range was set from 5 to 20 kDa for all study sample profiles. The m/z of each peak to be quantified (S/N ratio>5) was determined according to externally calibrated standards (Ciphergen Biosystems). The m/z sample peaks with more than 2000 m/z were normalized with Biomarker Wizard to compile all spectra and automatically detect quantified mass peaks.

### Bioinformatics and biostatistics

Serum samples were split into two groups, the training set or test set. Forty-five samples from ALL patients and 34 healthy controls were randomly selected for training sample set. To evaluate the accuracy and validity of the classification tree, 49 samples of ALL patients and 50 controls (20 healthy children and 30 patients with pediatric AML) were selected for the test set (Table 1).

The profiling spectra of serum samples from the training set were normalized using total ion current normalization by Ciphergen's ProteinChip Software (version 3.1). Peak labeling was performed by the Biomarker Wizard feature of the software. A two-sample *t*-test was used to compare mean normalized intensities between the case and control groups. The *p *value was set at 0.05 to be statistically significant. The intensities of selected peaks were then transferred to Biomarker Pattern Software (BPS) to construct the classification tree of ALL. Briefly, the intensities of the selected peaks were submitted to BPS as a 'Root note'. Based on peak intensity, a threshold was determined by BPS to classify the root node into two child nodes. If the peak intensity of a blind sample was lower than or equal to the threshold, this peak would be labeled as "left-side child node." Peak intensities higher than the threshold would be marked as "right-side child node." After rounds of decision making, the training set was found to be discriminatory with the least error.

All of the protein peak intensities of samples in the test set were evaluated by BPS using the classification model. The ALL and control samples were then discriminated based on their proteomic profile characteristics. The sensitivity was defined as the probability of predicting ALL cases, and the specificity was defined as the probability of predicting control samples. A positive predictive value reflected the probability of ALL if a test result was positive.

### Serum fractionation

Serum samples from both healthy controls and ALL patients were selected for the purification of the four candidate protein biomarkers. The serum sample was mixed with U9 buffer (1:2, v/v) and incubated for 30 min at room temperature. The sample was then diluted in 5 mL of WCX binding buffer (50 mM NaAc, pH 4.0) and loaded to the CM Ceramic Hyper D WCX SPE column (6 × 10 mm, Pall Life science, USA). After washing with 2 mL of WCX binding buffer, the column was eluted with 5 mL of eluting buffer (2 M NaCl, 50 mM NaAc, pH 4.0) at a flow rate of 0.5 mL/min. The eluted fraction was further purified using HPLC.

### Purification of candidate protein markers using HPLC

HPLC separation was performed using SCL-10AVP (Shimadzu, Japan) with a Sunchrom C18 column (250 × 4.6 mm, 5 μm particle size, 300 Å) (The Great Eur-Asia Sci-Tech Development Co. Ltd, Beijing, China) and a C18 guard column (10 × 3 mm, Shimadzu, Japan). The mobile phase consisted of solvent A (5% ACN, 0.1% TFA) and solvent B (90% ACN, 0.1%TFA). The HPLC separation was achieved with a linear solvent gradient: 100% A (0 min)-15% B (15 min)-65% B (65 min)-100% B (100 min) at a flow rate of 0.5 mL/min. The eluate emissions were detected at multiple wavelengths of 214, 254, and 280 nm. Each peak fraction was collected and concentrated using SpeedVac, and then analyzed using an AXIMA-CFRTM plus MALDI-TOF mass spectrometer (Shimadzu/Kratos, Manchester, UK) in linear mode to trace the candidate protein biomarkers with α-cyano-4-hydrorycinnamic acid (CHCA) as the matrix.

### Identification of candidate protein biomarkers by LC-MS/MS

In-solution digestion of each concentrated fraction, which contains one candidate protein biomarker, was performed with a standard protocol. Briefly, each fraction was dissolved in 25 mM NH4HCO3, reduced with 10 mM DTT for 1 hour, and alkylated by 40 mM iodacetamide in the dark for 45 minutes at room temperature. Then, 40 mM DTT was added to quench the iodacetamide for 30 min at room temperature. Protease K (0.1 μg, Promega Corporation, USA) was then added into the sample solution and incubated for 45 min at 37°C. The digestion was stopped by adding formic acid to a final concentration of 0.1%. The digested sample was loaded onto a homemade C18 column (100 mm ×100 μm) packed with Sunchrom packing material (SP-120-3-ODS-A, 3 μm) and followed by nano-LC-ESI-MS/MS analysis. The LTQ mass spectrometer was operated in the data-dependent mode in which first the initial MS scan recorded the mass to charge (m/z) ratios of ions over the mass range from 400–2000 Da. The five most abundant ions were automatically selected for subsequent collision-activated dissociation. All MS/MS data were searched against a human protein database downloaded from the NCBI database using the SEQUEST program (Thermo, USA).

### Confirmation of candidate protein biomarkers using ProteinChip immunoassays

To confirm the identity of the candidate protein biomarkers, specific antibodies (anti-PF4 rabbit antibody, ab49735; anti-NAP-2 mouse antibody, ab58142, anti-C3a mouse antibody, ab11872, all from ABcam) were applied to each spot of pre-activated PS20 ProteinChip arrays (Ciphergen Biosystems) and incubated overnight at 4°C in a humidity chamber. After blocking with BSA and rinsing, antibody-coated spots were incubated with 1.5 μL of serum samples and 3 μL of binding buffer (0.1 M Na_3_PO_4_, 0.5 M urea, 0.5% CHAPS, pH 7.2) for 90 min [24]. Spots were then washed with PBST (0.5% Triton X-100), PBS and deionized water twice before drying. SELDI-TOF-MS analysis was performed on a PBS-II ProteinChip reader with CHCA as the matrix.

## Results

### Serum protein profiles and data processing

All the serum samples were collected from the selected patients and controls in 2004, then distributed into 20 mL per tube, and stored at -80°C until use. The SELDI-TOF-MS experiments were finished within three months after the collection of all serum samples. No significant change of the protein markers was found during storage after SELDI-TOF-MS and MALDI-TOF-MS analysis.

**Figure 1:**
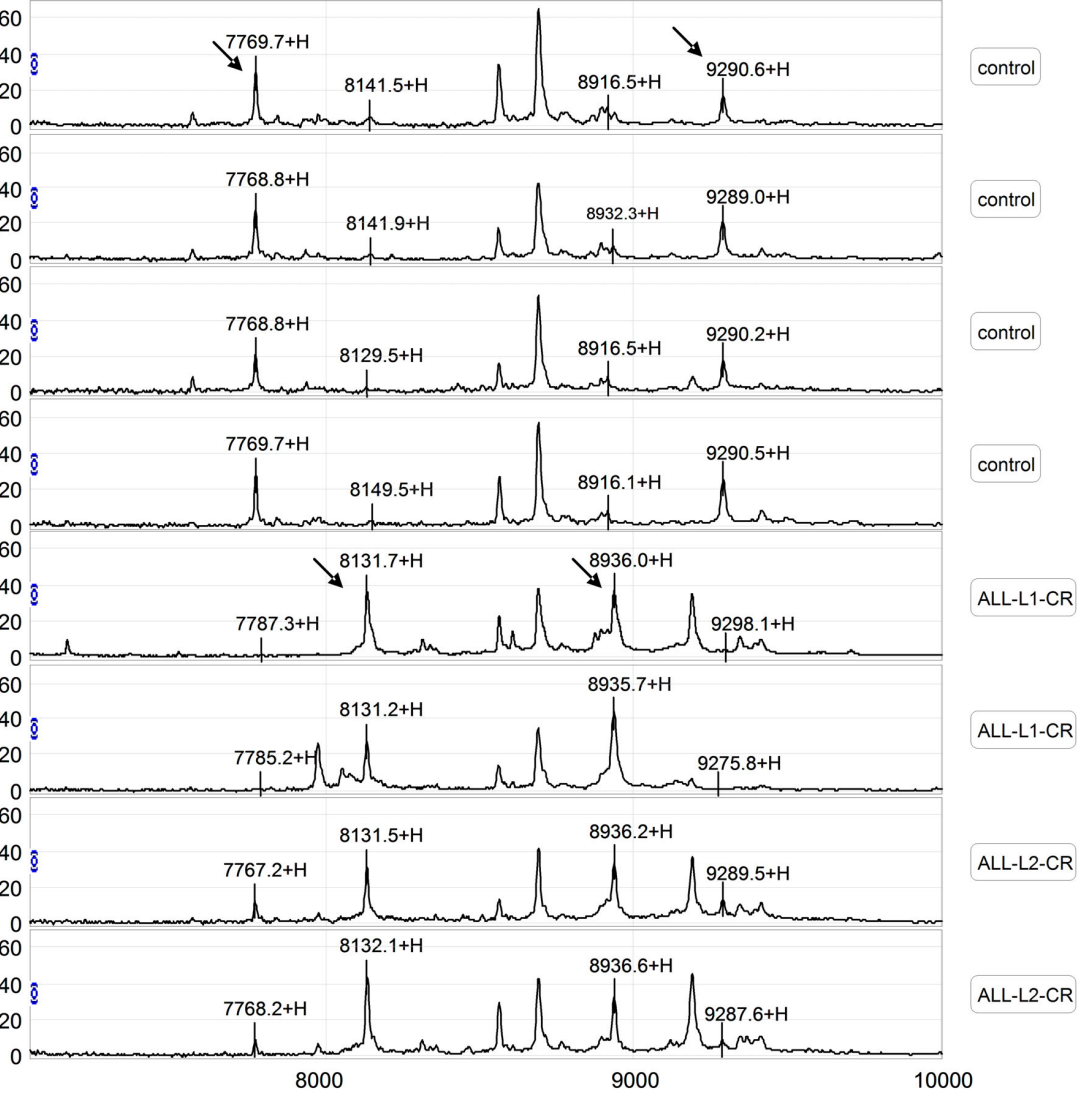
**Representative mapping of SELDI-TOF-MS analysis of sera from healthy controls and pediatric ALL patients**. Differentially expressed proteins with potential diagnostic significance are indicated by arrows. The top group denotes serum from a healthy volunteer, in which 7769 m/z and 9290 m/z were up-regulated. The bottom group denotes sera from patients with ALL, in which 8137 m/z and 8937 m/z were over-expressed.

Serum samples from the training set were evaluated by comparing the results obtained by SELDI-TOF-MS with those from the WCX2 chip. All MS data had baseline subtracted and were normalized using total ion current. Peak clusters were then generated by Biomarker Wizard software. Twenty-six peaks had statistically significant differences between pediatric patients and healthy children (*p *value < 0.05). In the ALL group, five protein peaks were found to be up-regulated and twenty-one peaks were found to be down-regulated (Table 2). Figure 1 shows the protein profiling patterns of sera from ALL patients and control samples. The results indicated that two protein peaks (8137 m/z, p value 6.07E-05 and 8937 m/z, p value 5.13E-05) were up-regulated in sera from ALL patients, while two other peaks (7769 m/z, p value 1.54E-07 and 9290 m/z, p value 7.59E-08) were down-regulated, compared with those from the healthy controls.

**Table 2:** The average peak intensity of proteins found in the sera of pediatric ALL patients.

m/z	Children with ALL		Healthy children		p value
		
	Mean	SD	Mean	SD	
9290.355	9.730	5.685	18.405	5.858	7.19E-08

7768.866	9.912	6.651	20.817	8.488	1.54E-07

15108.479	1.372	2.172	4.166	4.963	4.91E-07

7565.087	2.059	3.169	6.044	6.228	2.21E-06

4473.963	10.652	6.843	5.248	2.698	2.57E-05

8936.734	18.490	14.282	6.568	3.907	5.13E-05

8137.299	19.247	17.181	4.160	3.109	5.35E-05

28011.161	0.372	0.229	0.518	0.223	4.56E-04

15850.461	1.338	1.560	2.452	2.713	5.91E-04

5637.825	15.612	9.664	25.800	14.650	8.78E-04

4070.961	7.856	6.540	3.095	3.291	1.38E-03

3220.724	4.879	3.041	6.831	2.563	1.53E-03

4349.558	13.206	6.407	17.579	5.704	2.53E-03

6434.393	25.869	12.180	32.709	8.287	3.38E-03

23416.789	0.450	0.251	0.603	0.272	5.56E-03

4095.034	40.820	19.852	54.436	16.976	5.91E-03

2045.834	9.700	3.396	12.768	5.107	7.08E-03

7954.897	1.713	1.242	2.584	1.890	1.00E-02

2068.902	4.773	2.398	6.868	4.203	1.29E-02

2758.988	4.654	3.663	6.351	3.866	1.61E-02

4117.262	11.073	7.452	14.543	5.564	1.70E-02

5906.757	10.203	9.880	15.548	11.738	2.66E-02

8689.917	39.521	13.873	46.393	11.731	2.79E-02

4139.322	9.727	5.229	12.083	4.372	3.67E-02

6855.835	6.737	2.966	5.535	2.367	4.34E-02

8562.074	20.170	11.250	23.342	7.977	4.99E-02

### Protein peak detection and validation

**Figure 2:**
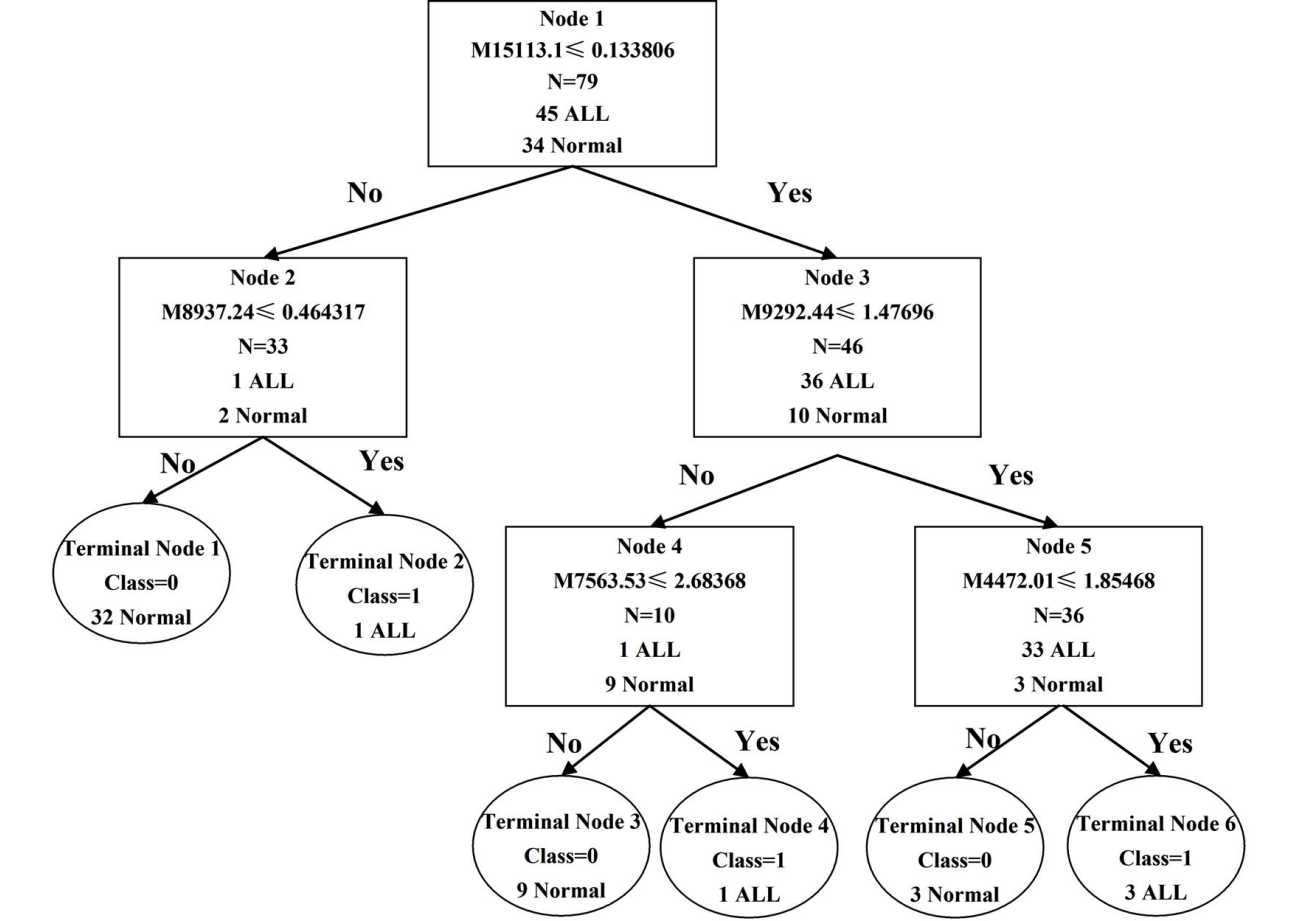
**Diagram of the classification tree for pediatric ALL patients and healthy controls**.

To develop biomarker patterns for the diagnosis of ALL, the intensities of the protein peaks in the training set were submitted to BPS. A total of seven peaks (9290 m/z, 7769 m/z, 15110 m/z, 7564 m/z, 4469 m/z, 8937 m/z, 8137 m/z) with the highest discriminatory power were automatically selected to construct a classification tree. Figure 2 shows the tree structure and sample distribution. The classification tree using the combination of the five peaks identified 45 ALL and 34 healthy subjects with a calculated sensitivity of 96% and a specificity of 98%.

To validate the accuracy and validity of the classification model derived from the training set, we applied the derived classification tree to a test dataset consisting of 49 ALL and 50 control samples. The classification tree discriminated the ALL samples from the controls with a sensitivity of 91.8% and a specificity of 90%. The positive predictive value was found to be 90%.

### Purification and identification of candidate protein biomarkers

**Figure 3:**
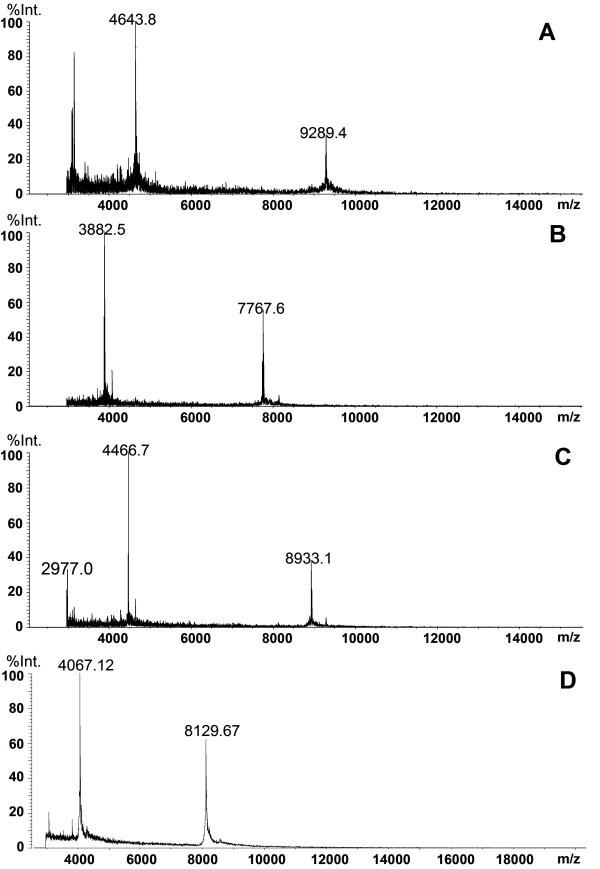
**MALDI-TOF-MS spectra of four purified potential protein markers**.

Serum samples from ALL patients were used for the purification of the two up-regulated candidate protein biomarkers (8137 and 8937 m/z), and serum samples from healthy controls were used for the purification of the two down-regulated proteins (7769, 9290 m/z) in the sera from ALL patients using WCX SPE and C18 HPLC. Figure 3 shows the results of MALDI-TOF-MS analyses of the four purified candidate protein biomarkers.

**Figure 4:**
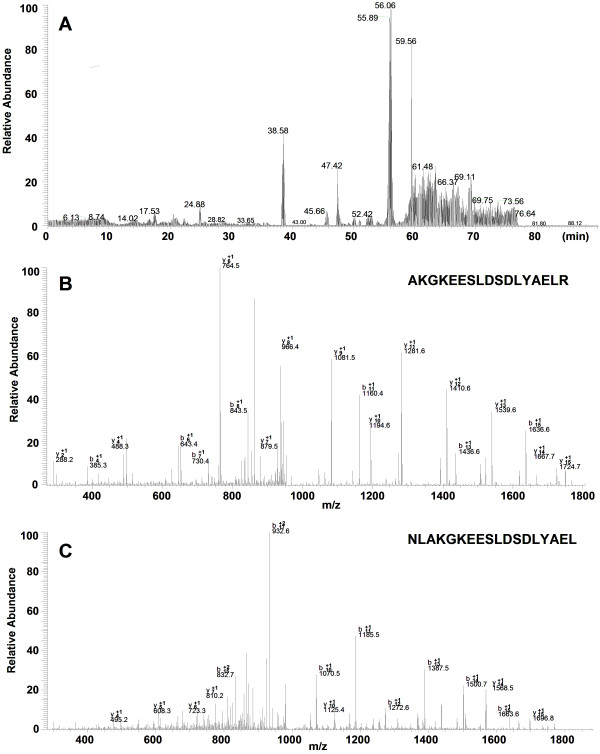
**Results of the identification of protein (9290 m/z) by LC-MS/MS**. (A) Chromatogram of peptide mixture; (B, C) MS/MS spectra of two peptides.

After digestion with protease K, the peptide mixture was analyzed by nano-LC-MS/MS. Figure 4 shows the results of the LC-MS/MS chromatogram (A) and MS/MS spectra of two identified peptides (B, C) from the protein (9290 m/z). Table 3 shows the results of the identification of the four candidate protein biomarkers: CTAP-III (fragment of pro-platelet basic protein precursor, PBP, 9290 m/z), PF4 (platelet factor 4, 7769 m/z), and two fragments of C3a (one of human complements, 8137 and 8937 m/z). A combination of high sequence coverage and accurate MW measurement by MALDI-TOF-MS provided a complete sequence of the four candidate protein markers.

**Table 3:** Identification of the four potential protein biomarkers with identified peptides and covered sequences.

*m/z*	*Protein name*	*Peptides identified*	Sequence*
9290	CTAP-III	K.RNLAKGKEESLDSDLYAELR.C	**rnlakgkeesldsdlyaelr**cmcikttsgihpkniqslevigkgthc**nqveviatlkdgrkicldpdaprikkivqkklagdesad**
			
		K.GKEESLDSDLYAEL.R	
			
		L.AKGKEESLDSDLYAELR.C	
			
		K.GKEESLDSDLYAELR.C	
			
		C.NQVEVIATLKDGRKICLDPDAPRIK.K	
			
		R.NLAKGKEESLDSDLYAELR.C	
			
		K.LAGDESAD.-	

7769	PF4	T.SQVRPRHITSL.E	eaeedgdlqclcvktt**sqvrprhitslevikagphcptaqliatlkngrkicldlqaplykkiikklles**
			
		T.SLEVIKAGPHCPTAQ.L	
			
		C.LDLQAPLYKKIIK.K	
			
		L.EVIKAGPHCPTAQ.L	
			
		L.DLQAPLYKKIIK.K	
			
		A.GPHCPTAQLIATLK.N	
			
		L.QAPLYKKIIKKLLES.-	

8137	C3a	R.SVQLTEKRMDKVGKYPKELRKC.C	**svqltekrmdkvgkypkelrkccedgmrenpmrfscq**rrtrfisl**geackkvfldccnyitelrrqha**
			
		E.ACKKVFLDCC.N	
			
		L.GEACKKVFLDCC.N	
			
		C.CEDGMRENPMRFSCQ.R	
			
		L.GEACKKVFLDCCNYITELRRQHA.R	

8937	C3a	R.SVQLTEKRMDKVGKYPKELRKC.C	**svqltekrmdkvgkypkelrkccedgmrenpmrfscq**rrtrfisl**geackkvfldccnyitelrrq**harashlgla
			
		E.KRMDKVGKYPKELRKC.C	
			
		C.CEDGMRENPMRFSCQ.R	
			
		L.GEACKKVFLDCC.N	
			
		T.EKRMDKVGKYPKELRKC.C	
			
		L.GEACKKVFLDCCNYITELRRQ.H	

*: Underlined and bold letters show the sequences corresponding to the identified peptides.

### Validation of four candidate protein biomarkers

**Figure 5:**
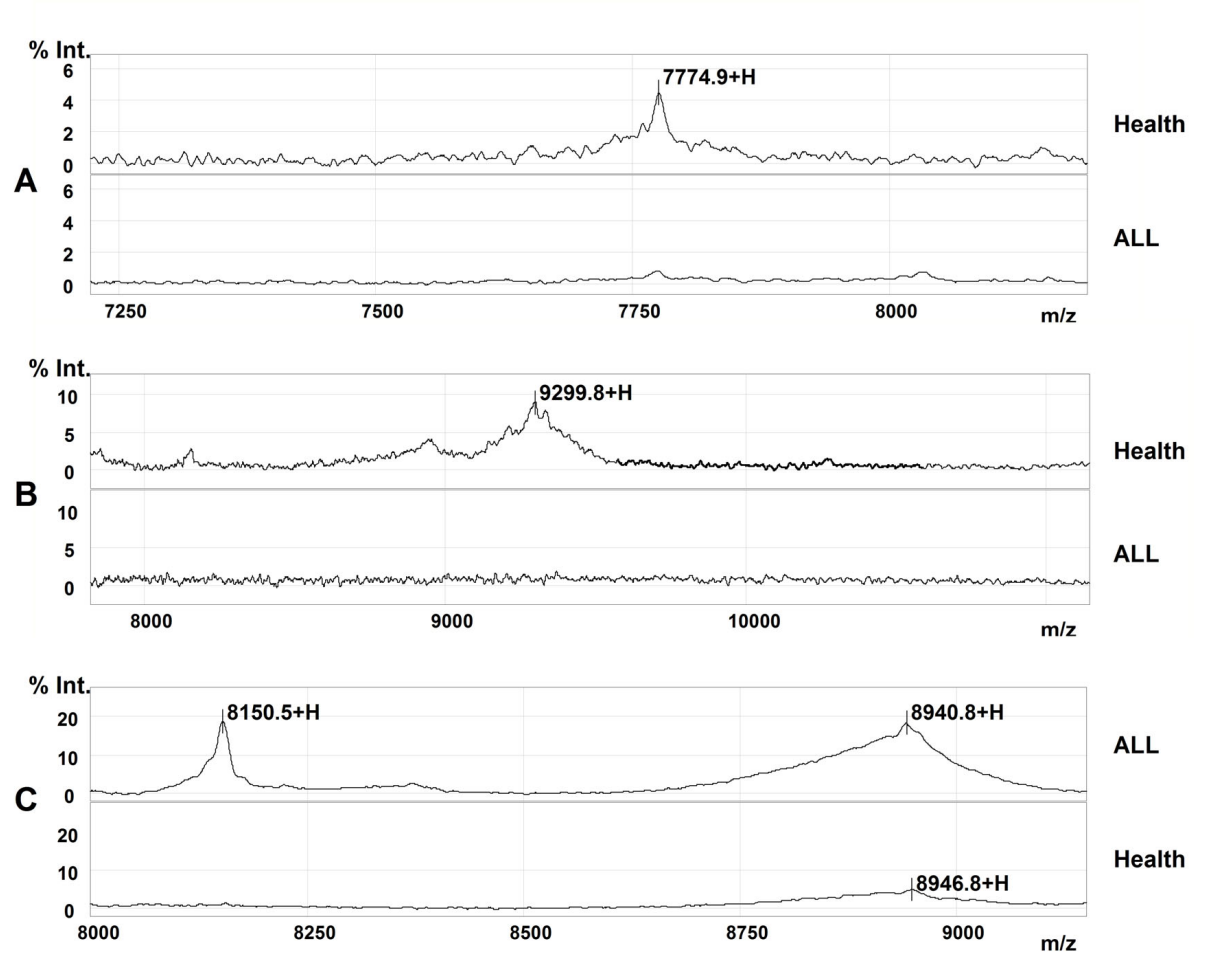
**Validation of four potential protein markers by SELDI immunoaffinity assay**. A: CTAP-III; B: PF4; C: fragments of C3a.

To confirm the identity of the four proteins as PF4, CTAPIII, and two fragments of C3a, we performed immunoassays with specific antibodies directed against the four proteins immobilized on a ProteinChip (Ciphergen Biosystems). The results showed that the CTAP-III and PF4 were captured and detected in the serum of healthy, but not ALL patients, controls, and the fragments of C3a in the serum of ALL patients, but not healthy controls (Figure 5).

## Discussion

In this study, we obtained serum protein mass spectra from pediatric ALL patients and controls using SELDI-TOF-MS. During the pilot study, we tried SCX2, WCX, IMAC-Cu, IMAC-Ni and H4 protein chips for the sera samples. Eventually, we found that more protein peaks were detected using the WCX protein chip to assay serum samples (data not shown in this paper.) Based on the serum proteomic profiles, we constructed a classification model to discriminate the ALL patients from the healthy controls. We used a two-step approach for proteomic biomarker screening. First, we employed a "training set" to identify differentially expressed proteins and used the distinguishing proteomic peaks from BPS for the construction of a classification tree. The classification model discriminated patients with ALL from healthy controls and pediatric AML with a sensitivity of 91.8% and a specificity of 90.0%.

Two down-regulated candidate protein biomarkers were identified as PF4 (platelet factor 4, 7769 m/z) and CTAP-III (fragment of pro-platelet basic protein precursor, PBP, 9290 m/z). Additionally, two up-regulated candidate protein biomarkers (8137 and 8937 m/z) were identified as two fragments of C3a.

Among the proteins identified by LC-MS/MS, both PF4 and CTAP-III are platelet-derived chemokines. PF4, which is also known as CXCL4, is present in platelet α-granules and is released during platelet aggregation. PF4 is also released from activated T lymphocytes and mast cells [25]. PF4 has been reported to influence many biological processes, including endothelial cell proliferation, migration, and angiogenesis [26-28]. PF4 can down-regulate cell proliferation and cytokine release, thereby inhibiting T cell functions [29], and could support the survival of normal hematopoietic precursors, protecting them from the toxicity of chemotherapeutic agents [30]. CTAP-III is an N-terminal cleavage product of PBP, which is synthesized by megakaryocytes. CTAP-III, β-thromboglobulin and NAP-2, which are derived from PBP through proteolysis, belong to a group of homologous and immunologically cross-reactive proteins derived from platelet α-granules. It was reported that CTAP-III could support stem cell-derived hematopoiesis [30]. Both PF4 and CTAP-III could protect early cells from the toxic effects of various chemotherapeutic agents [30]. Vermeulen found that average levels of PF4 and CTAP-III were down-regulated in the serum of benzene-exposed workers in comparison with control subjects [24].

Complement components play important roles as mediators of inflammation and immune responses. Complement 3, which is composed of α and β chains, is the most abundant complement component in serum (1.2 mg/mL). Complement 3 convertase could cleave C3 at the residues Arg726- Ser 727 giving rise to C3b (Mr 176000 Da) and C3a (Mr 9000 Da) [31]. C3a was reported to be an inflammatory mediator of innate immune responses [32], suggesting that C3a might represent an inflammation biomarker. C3a was found to be up-regulated in the ascitic fluids of ovarian cancer patients [33]. Lee found that C3a is elevated in patients with chronic hepatitis C and HCV-related hepatocellular carcinoma [34]. The 8.1 kDa C3a fragment was identified for the first time.

In summary, we identified a set of protein peaks that could discriminate pediatric ALL from healthy controls. From the protein peaks specific for pediatric ALL disease, we identified platelet factor (PF4), a fragment of pro-platelet basic protein precursor (CTAP-III) and C3a as potential proteomic biomarkers of pediatric ALL. This panel of markers is likely to be limited to distinguishing pediatric ALL from healthy controls and pediatric AML patients. Further studies with additional populations or using pre-diagnostic sera are needed to confirm the importance of these findings as diagnostic markers of pediatric ALL.

## Competing interests

The authors declare that they have no competing interests.

## Authors' contributions

YFQ and ZJZ designed the study. SLN was responsible for laboratory studies and drafted the manuscript. ZJ carried out the SELDI-TOF experiments. WP and LJ participated in the purification and identification of all the biomarkers. FK carried out the SELDI-TOF data analysis. XZS, XP and CTX participated in data analysis. CZY, CXL and HJJ participated in the design of the study and helped to draft the manuscript. All authors read and approved the final manuscript.

## References

[B1] Pui CH, Relling MV, Downing JR (2004). Acute lymphoblastic leukaemia. N Engl J Med.

[B2] Parker SL, Tong T, Bolden S, Wingo PA (1997). Cancer statistics, 1997. CA Cancer J Clin.

[B3] Cortes JE, Kantarjian HM (1995). Acute lymphoblastic leukemia. Cancer.

[B4] Pui CH, Behm FG, Crist WM (1993). Clinical and biologic relevance of immunologic marker studies in childhood acute lymphoblastic leukemia. Blood.

[B5] Pui CH (1995). Childhood Leukemias. New England Journal of Medicine.

[B6] Woolfson Adrian, Stebbing Justin, Tom BrianDM, Stoner KerrynJ, Gilks WalterR, Kreil DavidP, Mulligan StephenP, Belov Larissa, Chrisp JeremyS, Errington Will, Wildfire Adrian, Erber WendyN, Bower Mark, Gazzard Brian, Christopherson RichardI, Scott MikeA (2005). Conservation of unique cell-surface CD antigen mosaics in HIV-1-infected individuals. Blood.

[B7] Melhem R, Hailat N, Kuick R, Hanash SM (1997). Quantitative analysis of Op 18 phosphorylation in childhood acute leukemia. Leukemia.

[B8] Brattsand G (2000). Correlation of oncoprotein 18/stathmin expression in human breast cancer with established prognostic factors. Br J Cancer.

[B9] Chen G, Wang H, Gharib TG, Huang CC, Thomas DG, Shedden KA, Kuick R, Taylor JM, Kardia SL, Misek DE (2003). Overexpression of Oncoprotein 18 Correlates with Poor Differentiation in Lung Adenocarcinomas. Mol Cell Proteomics.

[B10] Paradis V, Degos F, Dargere D, Pham N, Belghiti J, Degott C, Janeau JL, Bezeaud A, Delforge D, Cubizolles M (2005). Identification of a new marker of hepatocellular carcinoma by serum protein profiling of patients with chronic liver diseases. Hepatology.

[B11] Ward DG, Cheng Y, N KG, Thar TT, Barget N, Wei W, Billingham LJ, Martin A, Beaugrand M, Johnson PJ (2006). Changes in the serum proteome associated with the development of hepatocellular carcinoma in hepatitis C-related cirrhosis. Br J Cancer.

[B12] Vlahou A, Schellhammer PF, Mendrinos S, Patel K, Kondylis FI, Gong L, Nasim S, Wright JG (2001). Development of a Novel Proteomic Approach for the Detection of Transitional Cell Carcinoma of the Bladder in Urine. Am J Pathol.

[B13] Adam BL, Qu Y, Davis JW, Ward MD, Clements MA, Cazares LH, Semmes OJ, Schellhammer PF, Yasui Y, Feng Z (2002). Serum Protein Fingerprinting Coupled with a Pattern-matching Algorithm Distinguishes Prostate Cancer from Benign Prostate Hyperplasia and Healthy Men. Cancer Res.

[B14] Cazares LH, Adam BL, Ward MD, Nasim S, Schellhammer PF, Semmes OJ, Wright JG (2002). Normal, Benign, Preneoplastic, and Malignant Prostate Cells Have Distinct Protein Expression Profiles Resolved by Surface Enhanced Laser Desorption/Ionization Mass Spectrometry. Clinical Cancer Research.

[B15] Petricoin EF, Ardekani AM, Hitt BA, Levine PJ, Fusaro VA, Steinberg SM, Mills GB, Simone C, Fishman DA, Kohn EC (2002). Use of proteomic patterns in serum to identify ovarian cancer. Lancet.

[B16] Petricoin EF, Ornstein DK, Paweletz CP, Ardekani A, Hackett PS, Hitt BA, Velassco A, Trucco C, Wiegand L, Wood K (2002). Serum Proteomic Patterns for Detection of Prostate Cancer. Journal of the National Cancer Institute.

[B17] Qu Y, Adam BL, Yasui Y, Ward MD, Cazares LH, Schellhammer PF, Feng Z, Semmes OJ, Wright JG (2002). Boosted Decision Tree Analysis of Surface-enhanced Laser Desorption/Ionization Mass Spectral Serum Profiles Discriminates Prostate Cancer from Noncancer Patients. Clinical Chemistry.

[B18] Lehrer S, Roboz J, Ding H, Zhao S, Diamond EJ, Holland JF, Stone NN, Droller MJ, Stock RG (2003). Putative protein markers in the sera of men with prostatic neoplasms. BJU International.

[B19] Kozak KR, Amneus MW, Pusey SM, Su F, Luong MN, Luong SA, Reddy ST, Farias-Eisner R (2003). Identification of biomarkers for ovarian cancer using strong anion-exchange ProteinChips: Potential use in diagnosis and prognosis. Proc Natl Acad Sci USA.

[B20] Shiwa M, Nishimura Y, Wakatabe R, Fukawa A, Arikuni H, Ota H, Kato Y, Yamori T (2003). Rapid discovery and identification of a tissue-specific tumor biomarker from 39 human cancer cell lines using the SELDI ProteinChip platform. Biochemical and Biophysical Research Communications.

[B21] Zhukov TA, Johanson RA, Cantor AB, Clark RA, Tockman MS (2003). Discovery of distinct protein profiles specific for lung tumors and pre-malignant lung lesions by SELDI mass spectrometry. Lung Cancer.

[B22] Albitar M, Potts SJ, Giles FJ, O BS, Keating M, Thomas D, Clarke C, Jilani I, Aguilar C, Estey E (2006). Proteomic-based prediction of clinical behavior in adult acute lymphoblastic leukemia. Cancer.

[B23] Hegedus CM, Gunn L, Skibola CF, Zhang L, Shiao R, Fu S, Dalmasso EA, Metayer C, Dahl GV, Buffler PA (2005). Proteomic analysis of childhood leukemia. Leukemia.

[B24] Vermeulen R, Lan Q, Zhang L, Gunn L, McCarthy D, Woodbury RL, McGuire M, Podust VN, Li G, Chatterjee N (2005). Decreased levels of CXC-chemokines in serum of benzene-exposed workers identified by array-based proteomics. Proc Natl Acad Sci USA.

[B25] Boehlen FCK (2001). Platelet chemokines and their receptors: what is their relevance to platelet storage and transfusion practice?. Transfus Med.

[B26] Strieter RM, Belperio JA, Phillips RJ, Keane MP (2004). Chemokines: Angiogenesis and metastases in lung cancer. Novartis Found Symp.

[B27] Szekanecz Z, Kim J, Koch AE (2003). Chemokines and chemokine receptors in rheumatoid arthritis. Seminars in Immunology.

[B28] Szekanecz Z, Koch AE (2001). Chemokines and angiogenesis. Current Opinion in Rheumatology.

[B29] Fleischer J, Grage-Griebenow E, Kasper B, Heine H, Ernst M, Brandt E, Flad HD, Petersen F (2002). Platelet Factor 4 Inhibits Proliferation and Cytokine Release of Activated Human T Cells 1. The Journal of Immunology.

[B30] Han ZC, Lu M, Li J, Defard M, Boval B, Schlegel N, Caen JP (1997). Platelet Factor 4 and Other CXC Chemokines Support the Survival of Normal Hematopoietic Cells and Reduce the Chemosensitivity of Cells to Cytotoxic Agents. Blood.

[B31] Sahu A, Lambris JD (2001). Structure and biology of complement protein C3, a connecting link between innate and acquired immunity. Immunological Reviews.

[B32] Markiewski MM, Mastellos D, Tudoran R, DeAngelis RA, Strey CW, Franchini S, Wetsel RA, Erdei A, Lambris JD (2004). C3a and C3b Activation Products of the Third Component of Complement (C3) Are Critical for Normal Liver Recovery after Toxic Injury 1. The Journal of Immunology.

[B33] Bjorge L, Hakulinen J, Vintermyr OK, Jarva H, Jensen TS, Iversen OE, Meri S (2005). Ascitic complement system in ovarian cancer. Br J Cancer.

[B34] Lee IN, Chen CH, Sheu JC, Lee HS, Huang GT, Chen DS, Yu CY, Wen CL, Lu FJ, Chow LP (2006). Identification of complement C3a as a candidate biomarker in human chronic hepatitis C and HCV-related hepatocellular carcinoma using a proteomics approach. Proteomics.

